# Characterization of the complete plastid genome of *Clivia mirabilis* (Amaryllidaceae)

**DOI:** 10.1080/23802359.2024.2444637

**Published:** 2024-12-23

**Authors:** Ling Yue, Xiu-Li Feng, Dan Li, Hai-Hong Wu, Jing Meng, Xing-Hua Zhao

**Affiliations:** a Institute of Floriculture, Liaoning Academy of Agricultural Sciences, Shenyang, China; b College of Landscape and Horticulture, Yunnan Agricultural University, Kunming, China

**Keywords:** Amaryllidaceae, *Clivia mirabilis*, phylogenetic analysis, plastid genome

## Abstract

*Clivia mirabilis* Rourke 2002 is an evergreen herbaceous flower with high ornamental value. In this study, we sequenced the complete chloroplast (cp) genome of *C. mirabilis* and reported it for the first time. The cp genome was 158,914 base pairs (bp) in total length, including two inverted repeats (IRs, 27,052 bp), separated by a large single-copy region (LSC, 86,519 bp) and a small single-copy region (SSC, 18,291 bp). There are 133 different genes in the cp genome of *Clivia mirabilis*, including 87 protein-coding genes, 38 transfer RNA genes, and eight ribosomal RNA genes. The overall GC content of the cp genome was 37.9%. Our phylogenetic analysis showed that *C. mirabilis* formed a monophyletic clade with the other sampled species of *Clivia*, falling into the Amaryllidoideae clade. Our findings could be used to identify and analyze the genetic diversity of *C. mirabilis* and provide new data for taxonomic and phylogenetic studies of *Clivia*.

## Introduction

*Clivia* Lindl., belonging to Amaryllidaceae, is an important ornamental potted flower, which is widely cultivated around the world (Koopowitz 2002). It is famous for its elegant and noble flower, long flowering period, evergreen herbaceous leaves and upright plant architecture. There are six species in *Clivia*, *C. miniata* (Lindl.) Verschaff., *C. nobilis* Lindl., *C. caulescens* R.A.Dyer*, C. gardenii* Hook., *C. mirabilis* and *C. robusta* Murray, et al. (Duncan 2008), among which *C. mirabilis* and *C. robusta* were newly discovered in 2002 and 2004, respectively (Rourke, 2002; Murray et al. 2004).

**Figure 1.**
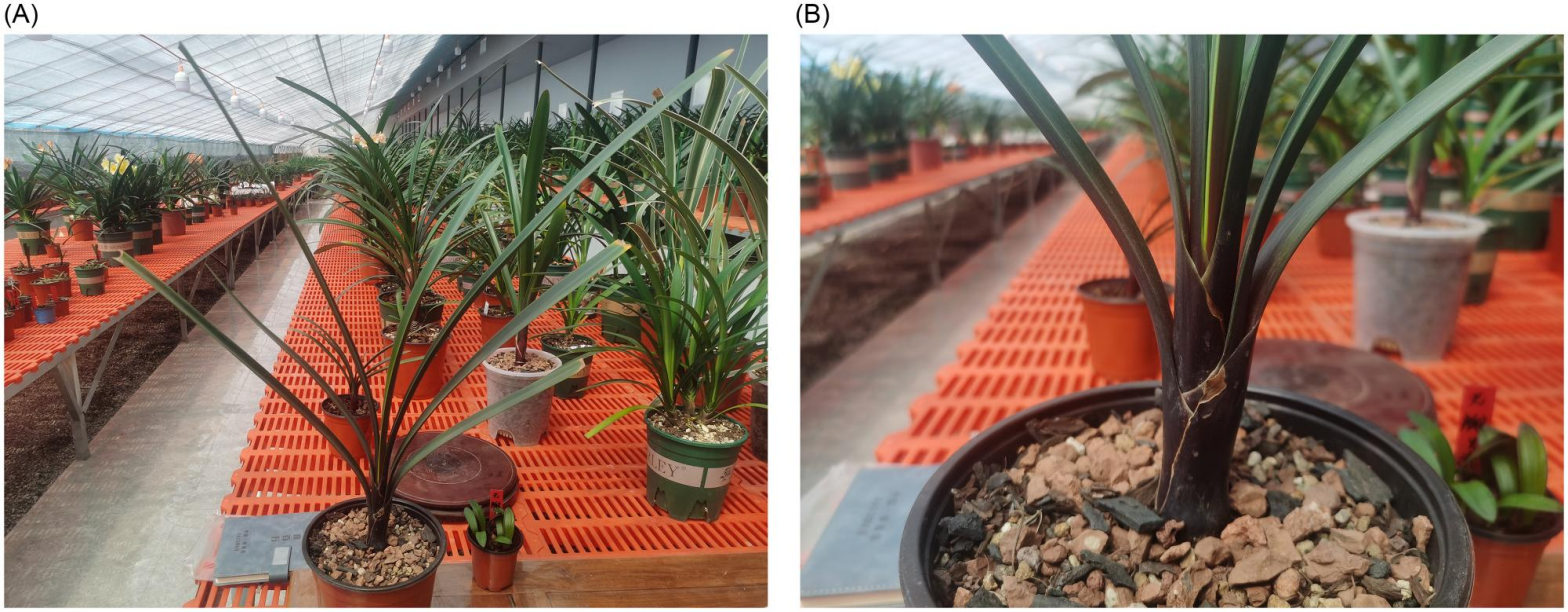
Morphology of *Clivia mirabilis*. (A) Individual of *Clivia mirabilis*; (B) Purplish red base of the leaves. This species is typically characterized by its light white stripe in the center of the leaves, a distinct purplish red base of the leaves, and a smooth leaf margin. The photos were taken by Ling Yue at the greenhouse of Liaoning Academy of Agricultural Sciences, Shenyang, China in may 2024, without any copyright issues.

Unlike the other five species distributed in the east and southeast of South Africa, *C. mirabilis* was found in the Oorlogskloof Nature Reserve, located southwest of South Africa. In addition, it is also the only one in *Clivia* distributed in the winter rainfall areas with an annual rainfall only about 410 mm. *Clivia mirabilis* grows at an altitude of 850-900 m and blooms from late October to late November. The most distinctive feature of *C. mirabilis* from the other *Clivia* species is the light white stripe in the center of the leaves, a distinct purplish red base of the leaves, and a smooth leaf margin (Figure 1). Its inflorescence is umbrella-shaped and pendulous, 20 to 48 flowers, with a purple to carmine peduncle. The flower color ranges from orange-yellow to orange-red, with a green tip that darkens when opened (Wang et al. 2023).

Assessing the population genetic diversity and demographic dynamics of wild germplasm resources is important for the future breeding of *Clivia*. Here, we report the first complete chloroplast genome assembly of *C. mirabilis,* which will support further phylogeographic and population genetic studies of this species.

## Materials and methods

Young fresh leaves of *C. mirabilis* were collected from Liaoning Academy of Agricultural Sciences (Shenyang, China; 41°48′33″N, E12°34′53″E) (Figure 1). Total genomic DNA was isolated following the modified CTAB method for constructing a 400 bp shotgun library, and a specimen was deposited at the Institute of Floriculture of Liaoning Academy of Agricultural Sciences (www.laas.cn, contact person Ling Yue, and email yueling801228@163.com) under the voucher number JZL010. Total genomic DNA was extracted using a modified CTAB method (Doyle and Doyle 1987). Reads of the plastid genome were assembled using CLC Genomic Workbench v10 (CLC Bio., Aarhus, Denmark). All the contigs were checked against the reference genome of *Clivia robusta* (MW660367) using BLAST (https://blast.ncbi.nlm.nih. gov/) and aligned contigs were oriented according to the reference genome. The complete plastid genomes were then constructed using Geneious v4.8.5 (Biomatters Ltd., Auckland, New Zealand) and were automatically annotated using DOGMA (http://dogma.ccbb.utexas.edu/). The Chloroplast Genome Viewer (CPGView) was used to test the accuracy of genome annotation and created the genomic circle map of *C. mirabilis* (http://www. 1kmpg.cn/cpgview) and the cis-splicing genes and trans-splicing genes were processed using CPGview (Liu et al. 2023). The complete chloroplast genome sequence of *C. mirabilis* was submitted to the GenBank database of the National Center for Biotechnology Information (NCBI) under accession number PP836161.

To identify the phylogenetic position of *C. mirabilis*, 25 plastid genomes downloaded from NCBI GenBank were aligned using the online program MAFFT (https://mafft.cbrc.jp/ alignment/server/index/index.html) (Katoh and Standley 2013), and the maximum likelihood (ML) tree was then conducted by PhyloSuite v1.2.3 (Zhang et al. 2020). Visualizing, modifying, and annotating phylogenetic trees is used by tvBOT, a user-friendly and efficient web application (Xie et al. 2023).

## Results

**Figure 2.**
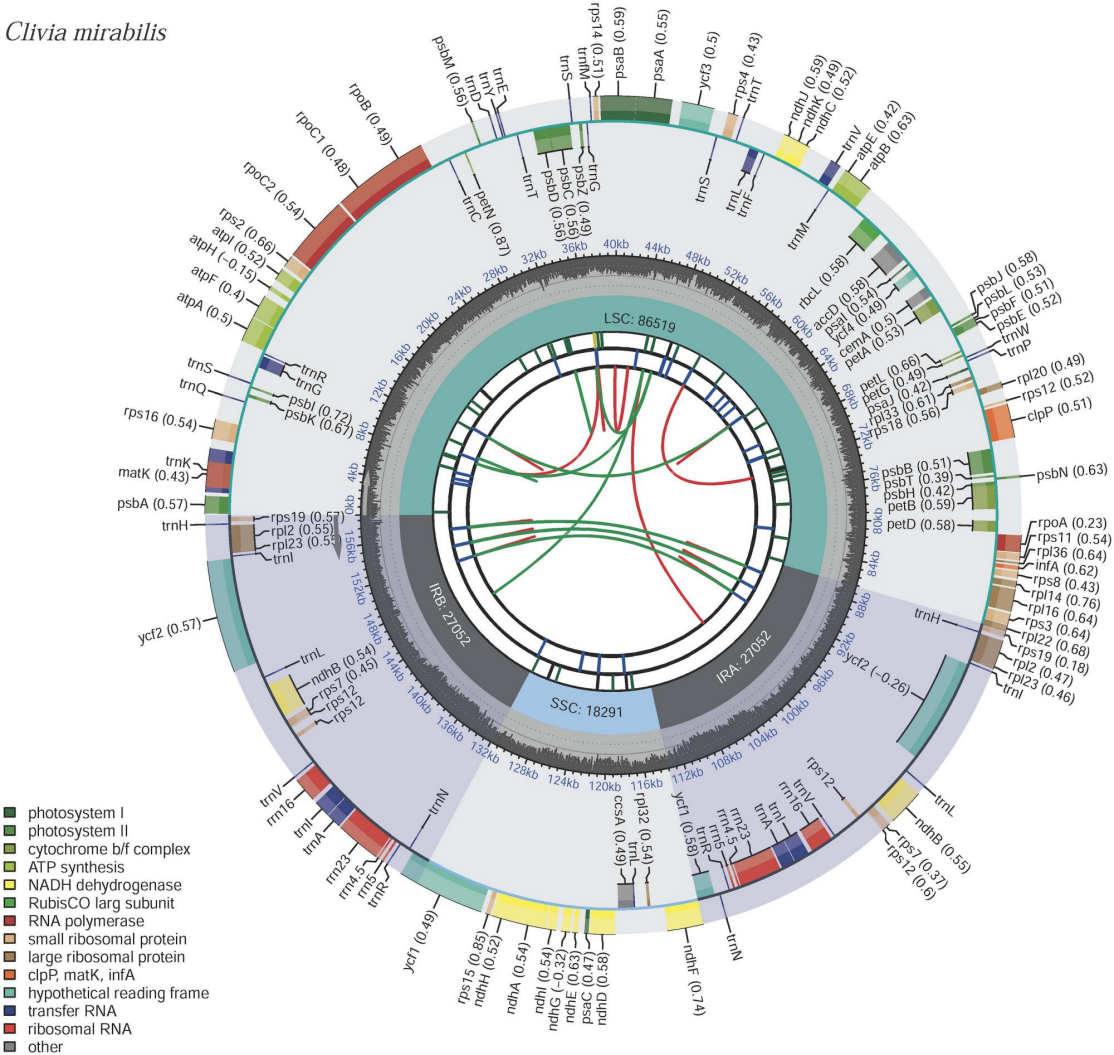
Gene maps of *C. mirabilis* genes lying outside the circle are transcribed in a clockwise direction, whereas genes on the inside are transcribed in a counterclockwise direction. Different colors denote known functional groups. The relative GC contents of genomic regions are represented in the inner circle by light gray. LSC, SSC, and IR indicate large single-copies, small single-copies, and inverted repeat regions, respectively.

A total of 4.09 Gb of raw data was produced by next-generation sequencing (NGS), and the average read mapping depth of the assembled genome was 558 X (Figure S1). The clean data of 3.93 Gb was retained after filtering reads with low quality. A total of 27,086,954 reads were produced with Q20 up to 97.34%. The reads were then used for *de novo* assembly of the chloroplast genome. The complete plastid genome of *C. mirabilis* represents a typical quadripartite circular molecule with 158,914 bp in length. It is composed of an LSC region of 86,519 bp, an SSC region of 18,291 bp and a pair of IR regions of 27,052 bp. The overall GC content of *C. mirabilis* plastid genome is 37.9% (Table S1). The GC content of the IR, SSC, and LSC regions of the genome was 42.9%, 32.1%, and 36.0%, respectively. The genome encodes 133 genes, including 87 PCG genes, 38 tRNA genes, and eight rRNA genes (Figure 2, Table S1). According to their function, all annotated genes were divided into four main categories, and most of the genes occurred in a single copy (Table S2). Ten genes (*rps16*, *atpF*, *ycf3*, *clpP*, *petB*, *petD*, *rpl16*, *rpl2*, *ndhA* and *rpl2*) and *ndhB* (two copies) were identified with a cis-spliced structure (Figure S2). The *rps12* was identified as a trans-spliced gene, containing three exons, with one being located in the LSC region and the other two in the IR regions (Figure S3).

**Figure 3.**
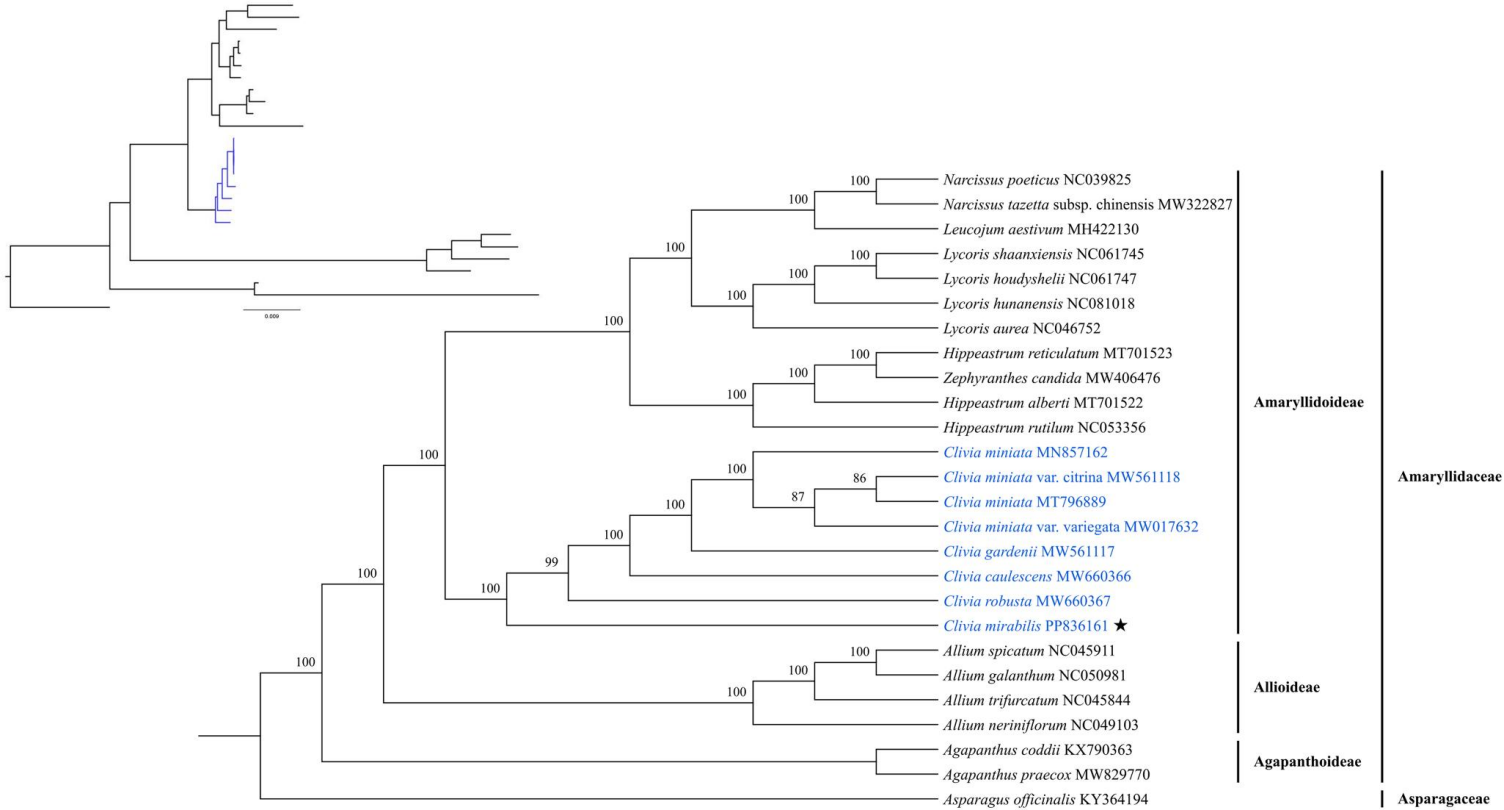
The maximum likelihood (ML) phylogenetic tree for *Clivia mirabilis* is based on 26 complete cp genomes. All sequences were downloaded from the NCBI GenBank database. Bootstrap support values (%) are indicated in each node. The star symbol indicates the sequence of cp genome for *Clivia mirabilis*. The following sequences were used: *Narcissus poeticus* NC039825 (Könyves et al. 2018), *Leucojum aestivum* MH422130 (Li et al. 2018), *Lycoris shaanxiensis* NC061745, *Lycoris houdyshelii* NC061747 (Zhang et al. 2021), *Lycoris aurea* NC046752 (Peng et al. 2020), *Hippeastrum reticulatum* MT701523, *Hippeastrum alberti* MT701522 (Liu et al. 2022), *Hippeastrum rutilum* NC053356 (He et al. 2021), *Clivia miniata* MN857162 (Wang et al. 2020), *Clivia miniata* var. *citrina* MW561118 (Zhao et al. 2022), *Clivia miniata* MT796889, *Clivia miniata* var. *variegata* MW017632 (Wu et al. 2023b), *Clivia gardenii* MW561117 (Wu et al. 2023a), *Clivia caulescens* MW660366 (Wu et al. 2021), *Clivia robusta* MW660367 (Zhao et al. 2022), *Allium spicatum* NC045911 (Yang et al. 2020), *Allium galanthum* NC050981 (Yusupov et al. 2021), *Allium trifurcatum* NC045844 (Yang et al. 2020), *Allium neriniflorum* NC049103 (Xie et al. 2020), *agapanthus praecox* MW829770 (Dong et al. 2021).

A phylogenetic tree was constructed using full-length chloroplast genome sequences, using seven published plastid genomes of the genus *Clivia* and seventeen species representing the seven genera from Amaryllidaceae, with *Asparagus officinalis* (KY364194) in Asparagaceae as the outgroup (Figure 3). The ML tree showed that the total 25 accessions from Amaryllidaceae clustered together, forming three clades representing the three subfamilies, Amaryllidoideae, Allioideae and Agapanthoideae. All of the accessions from *Clivia* formed an independent clade with a 100% bootstrap support. In addition, the *Clivia* clade clustered with all of the other species from Amaryllidoideae, including *Lycoris*, *Narcissus*, *Leucojum*, *Hippeastrum* and *Zephyranthes* with high support value.

## Discussion and conclusion

In this study, the complete chloroplast genome of *C. mirabilis* was first reported and analyzed. Comparing sequencing data with the four published *Clivia* species, we found that the full length of the chloroplast genomes in *Clivia* was highly conserved, with that of *C. mirabilis* being the longest (158,914 bp), and *C. robusta* the shortest (157,130 bp) (Table S1). Furthermore, the overall GC content (37.9%) was highly similar to that of the four published *Clivia* species (Table S1). Our phylogenetic analysis showed that all of the sampled species in the genus *Clivia* formed a monophyletic clade with high confident support. Furthermore, *Clivia* fell into the Amaryllidoideae clade, which was in accordance with the previously results (Wang et al. 2020; Wu et al. 2021, 2023a; Zhao et al. 2022). However, *Zephyranthes candida* nested with the *Hippeastrum* clade, and Zhao et al. (2022) also found the same results in the ML tree, indicating a closed relationship between these two genera. Finally, these findings above improve our understanding of the characteristics of the chloroplast genome of *C. mirabilis* and are valuable for future breeding and research efforts in *Clivia*.

## Supplementary Material

Supplementary materials.docx

## Data Availability

The genome sequence data that support the findings of this study are openly available in GenBank of NCBI at https://www.ncbi.nlm.nih.gov/ under accession no. PP836161. The associated BioProject, SRA, and Bio-Sample numbers are PRJNA1123403, SRR29383362, and SAMN41809875, respectively.
