# Supplementary material for: Characterization of the complete plastid genome of Clivia mirabilis (Amaryllidaceae)
Source: Mitochondrial DNA B Resour. 2024 Dec 23;10(1):42–6. doi: 10.1080/23802359.2024.2444637 (PMC11703478; doi:10.1080/23802359.2024.2444637)
Supplement: Supplementary materials.docx [file TMDN_A_2444637_SM2993.docx]

| Characteristics | *C. mirabilis* | *C. miniata* | *C. caulescens* | *C. robusta* | *C. gardenii* |
| --- | --- | --- | --- | --- | --- |
| Total in length(bp) | 158,914 | 158,114 | 158,149 | 157,130 | 158,156 |
| A large single copy region (LSC) in length (bp) | 86,519 | 86,204 | 86,250 | 85,430 | 86,307 |
| A small copy region (SSC) in length (bp) | 18,291 | 18,834 | 18,343 | 18,278 | 18,231 |
| Inverted repeat regions (IRa/IRb) in length (bp) | 27,052 | 26,788 | 26,778 | 26,711 | 26,809 |
| total GC% | 37.90 | 37.97 | 37.91 | 38.01 | 37.96 |
| Number of genes | 133 | 133 | 128 | 128 | 130 |
| Number of coding-protein | 87 | 87 | 86 | 86 | 86 |
| Number of rRNA | 8 | 8 | 8 | 8 | 8 |
| Number of tRNA | 38 | 38 | 34 | 34 | 36 |
| Gene Bank |  | MN857162 | MW660366 | MW660367 | MW561117 |
| Literature |  | Wang et al., 2020 | Wu et al., 2021 | Zhao et al., 2022 | Wu et al., 2023 |

Table S1 Summary of the sequencing data for five *Clivia* species

| Category | Gene groups | Gene names | Gene Num. |
| --- | --- | --- | --- |
| Photosynthesis | Subunits_of_photosystem_I | *psaA, psaB, psaC, psaI, psaJ* | 5 |
|  | Subunits_of_photosystem_II | *psbA, psbB, psbC, psbD, psbE, psbF, psbH, psbI, psbJ, psbK, psbL, psbM, psbN, psbT, psbZ, ycf3^b^* | 16 |
|  | Subunits_of_NADH_dehydrogenase | *ndhA^a^, ndhB^a^, ndhB^a^, ndhC, ndhD, ndhE, ndhF, ndhG, ndhH, ndhI, ndhJ, ndhK* | 12 |
|  | Subunits_of_cytochrome_b/f_complex | *petA, petB^a^, petD^a^, petG, petL, petN* | 6 |
|  | Subunits_of_ATP_synthase | *atpA, atpB, atpE, atpF^a^, atpH, atpI* | 6 |
|  | Large_subunit_of_Rubisco | *rbcL* | 1 |
| Self-replication | Large_subunits_of_ribosome | *rpl14, rpl16^a^, rpl2^a^, rpl20, rpl22, rpl23, rpl23, rpl2^a^, rpl32, rpl33, rpl36* | 11 |
|  | Small_subunits_of_ribosome | *rps11, rps12 ^b^, rps12 ^b^, rps14, rps15, rps16 ^a^, rps18, rps19, rps19, rps2, rps3, rps4, rps7, rps7, rps8* | 15 |
|  | DNA-dependent_RNA_polymerase | *rpoA, rpoB, rpoC1, rpoC2* | 4 |
|  | Ribosomal_RNAs | *rrn16, rrn16, rrn4.5, rrn4.5, rrn5, rrn5, rrn23, rrn23,* | 8 |
|  | Transfer_RNAs | *trnA-UGC^a^, trnA-UGC^a^, trnC-GCA, trnD-GUC, trnE-UUC, trnF-GAA, trnG-GCC, trnG-UCC^a^, trnH-GUG, trnH-GUG, trnI-CAU, trnI-CAU, trnI-GAU^a^, trnI-GAU^a^, trnK-UUU^a^, trnL-CAA, trnL-CAA, trnL-UAA^a^, trnL-UAG, trnM-CAU, trnN-GUU, trnN-GUU, trnP-UGG, trnQ-UUG, trnR-ACG, trnR-ACG, trnR-UCU, trnS-GCU, trnS-GGA, trnS-UGA, trnT-GGU, trnT-UGU, trnV-GAC, trnV-GAC, trnV-UAC^a^, trnW-CCA, trnY-GUA, trnfM-CAU* | 38 |
| Biosynthesis | Maturase | *matK* | 1 |
|  | Protease | *clpP^b^* | 1 |
|  | Envelope_membrane_protein | *cemA* | 1 |
|  | Acetyl-CoA_carboxylase | *accD* | 1 |
|  | C-type_cytochrome_synthesis_gene | *ccsA* | 1 |
|  | Translation_initiation_factor | *infA* | 1 |
| Genes of unknown | Proteins_of_unknown_function | *ycf1, ycf1, ycf2, ycf2, ycf4* | 5 |

Table S2 List of annotated genes in the chloroplast genomes of *C. mirabilis*

^a^ genes with one intron; ^b^ genes with two introns


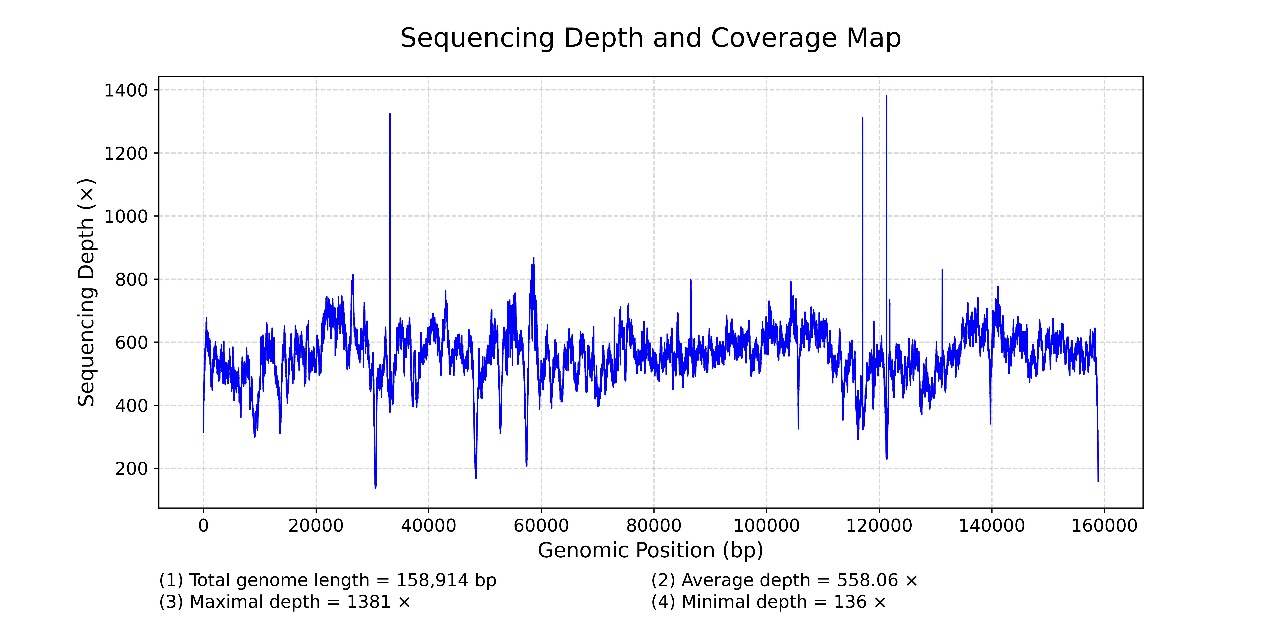


Figure S1 The read coverage depth map of the assembled genome


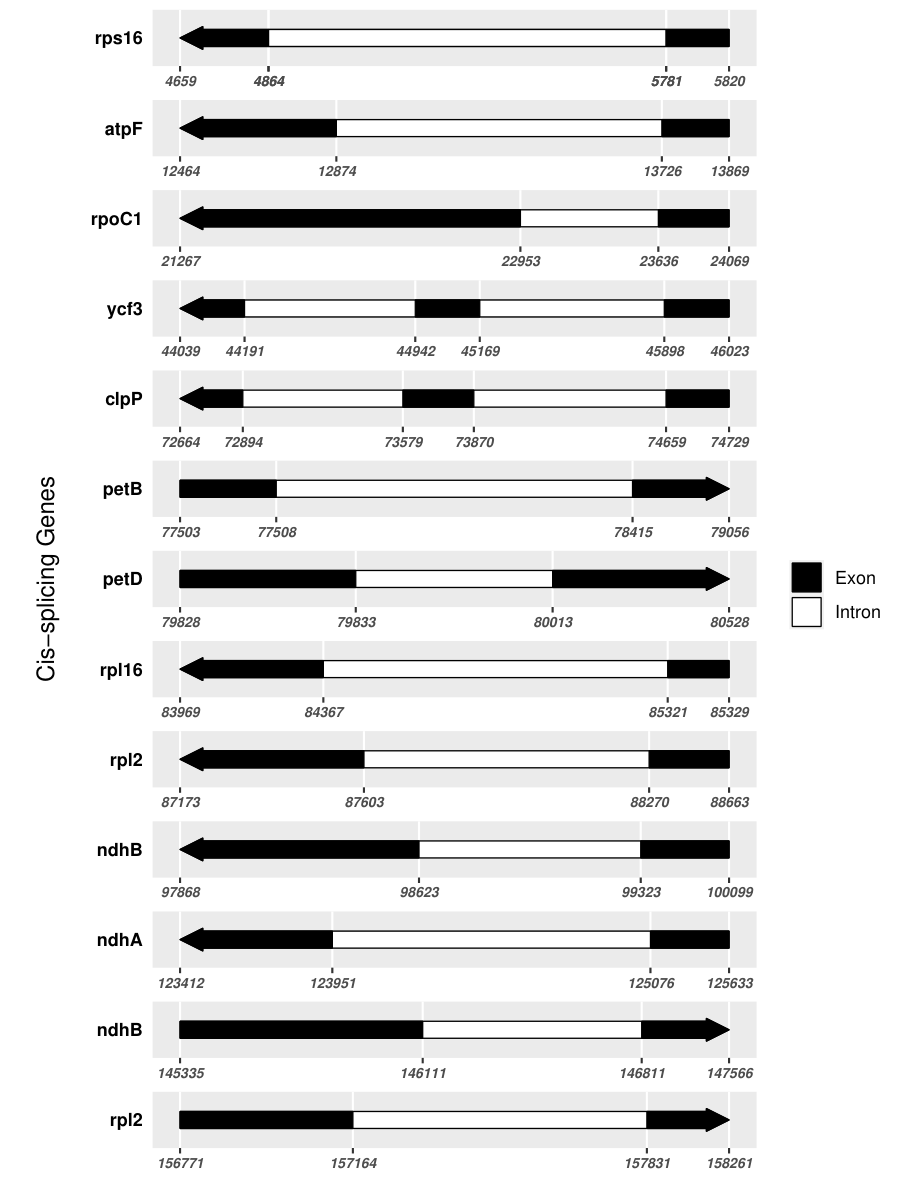
 Figure S2 The schematic map of the cis-splicing genes annotated in cp genome of *C. mirabilis*. (The numbers in the picture represent the location in the cp genome of *C. mirabilis*)


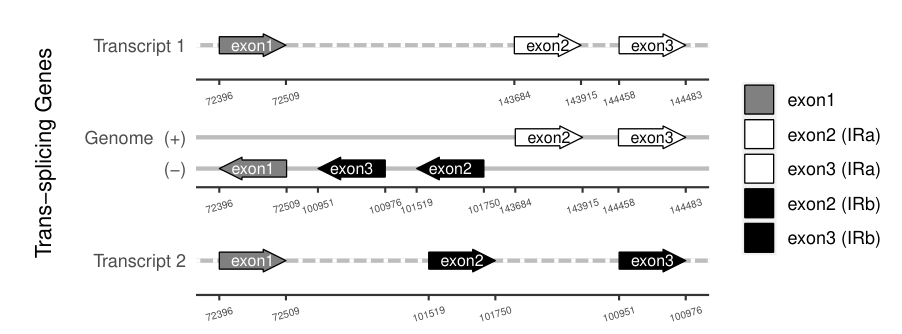


Figure S3 The schematic map of the trans-splicing gene *rps12* annotated in cp genome of *C. mirabilis*. (The numbers in the picture represent the location in the cp genome of *C. mirabilis*)
